# Early Permian terrestrial apex predator regurgitalite indicates opportunistic feeding behaviour

**DOI:** 10.1038/s41598-025-33381-0

**Published:** 2026-01-30

**Authors:** Arnaud Rebillard, Andréas Jannel, Lorenzo Marchetti, Mark J. MacDougall, Christopher Hamann, J.-Sébastien Steyer, Jörg Fröbisch

**Affiliations:** 1 Museum für Naturkunde Leibniz-Institut für Evolutions- und Biodiversitätsforschung, Invalidenstraße 43, 10115 Berlin, Germany; 2 Institut für Biologie, Humboldt-Universität zu Berlin, Invalidenstraße 42, 10115 Berlin, Germany; 3 Centre de Recherches en Paléontologie- Paris, UMR 7207, CNRS-MNHN-SU, Muséum National d’Histoire Naturelle, 8 Rue Buffon, CP38, 75005 Paris, France; 4 Liverpool John Moores University, James Parsons Building, 3 Byrom St, Liverpool, L3 3AF UK; 5 Brandon University, 270-18th Street, Brandon, MB R7A 6A9 Canada

**Keywords:** Ecology, Ecology, Evolution, Zoology

## Abstract

Fossilised digestive remains (bromalites) provide unique insights into extinct animals’ behavioural ecology, physiology and diet. We describe fossilised regurgitated stomach content from the early Permian Bromacker locality (Thuringia, Germany) using micro-CT, osteological, chemical and taphonomical analyses. The regurgitalite consists of a compact cluster of 41 bones with a unique taphonomic signature, including sub-articulated, aligned long bones, an irregular overall shape, and low phosphorus contents in the near-bone matrix. The multitaxic elements comprise a maxilla attributed to the captorhinomorph *Thuringothyris mahlendorffae*, postcranial elements of the bolosaurid *Eudibamus cursoris* and an unidentified diadectid, along with several unassignable elements, indicating opportunistic feeding behaviour. The regurgitalite size and composition suggest an apex predator as producer, such as the sphenacodontid *Dimetrodon teutonis* or the varanopid *Tambacarnifex unguifalcatus*, both known from Bromacker. This specimen represents the geologically oldest terrestrial regurgitalite and reveals novel insights into the feeding behaviours and the trophic network in a late Palaeozoic continental ecosystem.

**Supplementary Information:**

The online version contains supplementary material available at 10.1038/s41598-025-33381-0.

## Introduction

Bromalites comprise all fossil specimens of digestive origin, including faeces (i.e., coprolites), material preserved inside the digestive tract (e.g., consumulites, cololites) or remains that were regurgitated (i.e. regurgitalites)^1^. Even if rarely identified in the fossil record, regurgitation is a very common behaviour in vertebrates, amongst both carnivores and herbivores, brought to the extreme in the digestive process of ruminants. Gastric egestion, representing the oral ejection of digestive material, is also a biological mechanism common in vertebrates, but in particular in carnivorous taxa, as the consumed prey contains tissues that are noxious or not easily digestible such as bones, skin, scales, hair and feathers^2^. These elements can be orally expelled as compact ejecta and this content of regurgitation may be cemented together with gastric secretions, making it highly resilient to environmental degradation^2–4^. Whereas faecal matter passes through the entire digestive system, regurgitation occurs at an earlier stage of digestion, resulting in differences of morphology and key characteristics^5,6^. As with coprolites^7,8^, regurgitalites can have an exceptional fossil preservation potential^9^, primarily due to their high degree of structural cohesion^2,10^. This cohesion may allow for the preservation of more fragile structures, such as muscle tissues^9^, highlighting the potential and importance of correctly identifying such bromalites. Palaeozoic regurgitalites are so far only known from aquatic depositional environments, such as marine, lagoonal and lacustrine settings^1,11^. These deposits provide more optimal conditions for fossilisation than terrestrial ones due to higher sedimentation rate and calmer settings. Here, we describe a bone cluster (MNG 17001) from the famous early Permian Bromacker locality^12,13^ of central Germany that we interpret as a regurgitalite, using micro x-ray computed tomography (µCT) as well as osteological, chemical and taphonomical analyses. This find represents the geologically oldest regurgitalite from a terrestrial palaeoenvironment and provides unique insights into the feeding ecology of late Palaeozoic tetrapods.

## Geological setting

The Bromacker locality has been interpreted as one of the few fully terrestrial early Permian vertebrate assemblages currently known^12,13^. It belongs to the terrestrial Tambach Formation, part of the Thuringian Forest Basin, situated in Thuringia, Germany. The Tambach Formation is a 200–280 m thick unit including conglomerates, sandstones and mudstones, representing an alluvial fan setting, including fluvial and floodplain depositional environments^14^. Fossils are only known from the Tambach Sandstone Member, and almost entirely from the Bromacker locality^15^. It is a relatively small area including a few quarries about 2 km north of the village of Tambach-Dietharz. This locality is famous for its unique and diverse faunal assemblage, dominated by herbivorous diadectids^13,16–18^. The vertebrate component of the assemblage further consists of various terrestrial anamniotes, synapsids and reptiles as well as tetrapod footprints^19,20^, resting traces^21^ and burrows^22^. The age of the Tambach Formation, based on the fossil content and on U-Pb radiometric dating on volcanic units in the underlying formations, is probably Sakmarian^23,24^.

**Fig. 1:**
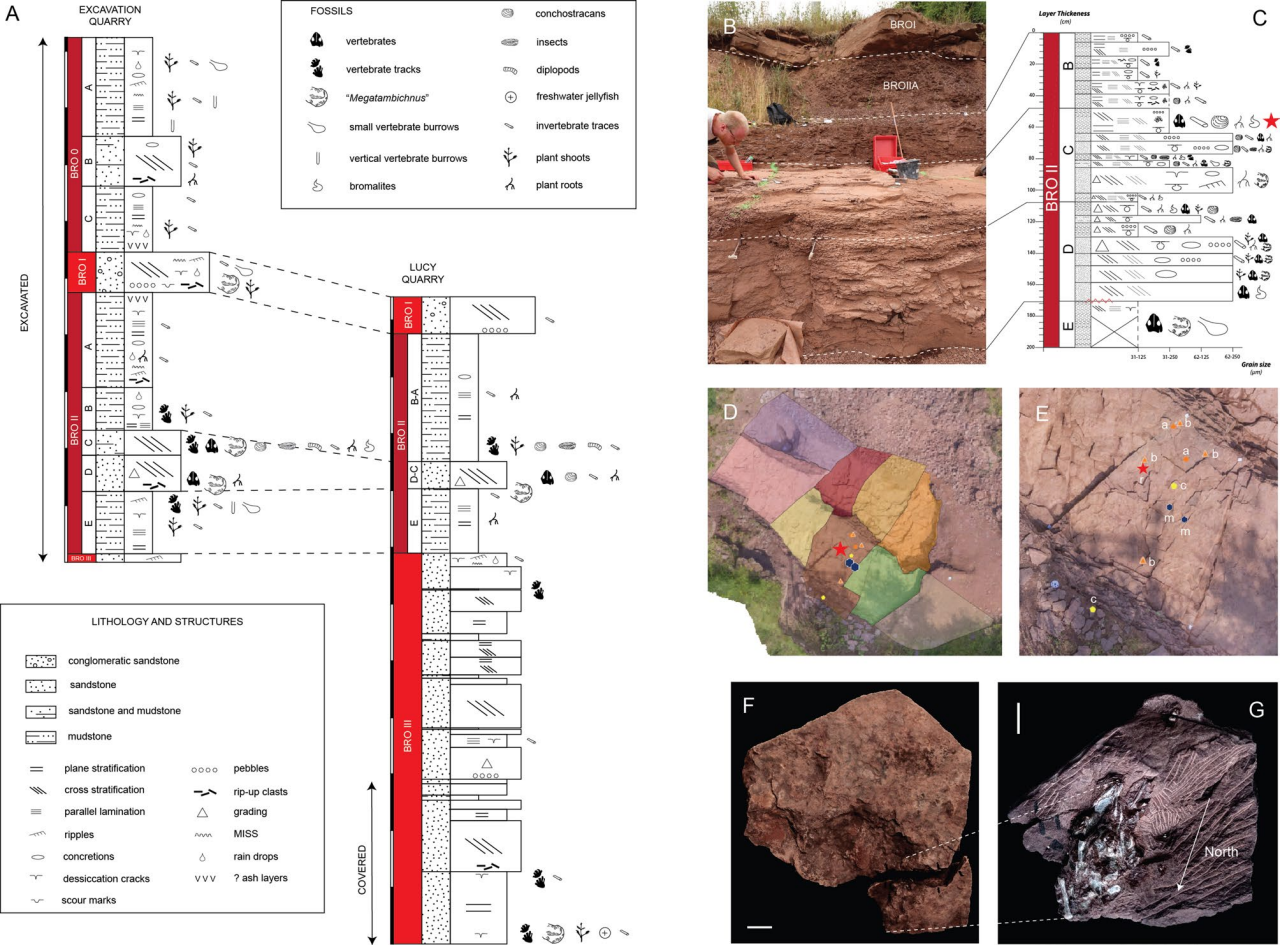
Stratigraphy of the Bromacker locality and GPS location and orientation of MNG 17001. (**A**) Stratigraphic log of the Bromacker locality (Tambach Formation, early Permian, Thuringia, Germany). (**B**) Stratigraphy of the sector WE3. (**C**) Stratigraphic log of the sector WE3. The star indicates MNG 17001. (**D**) Orthophotograph of the excavated western area of the quarry and sectors evidenced with different colours. The image was generated using QGIS Desktop 3.34.14 (https://qgis.org/), and GPS coordinates were obtained using a georeferenced total station (Leica Flexline TS06 Plus). (**E**) Enlargement of D, showing the GPS location of the fossil findings of WE3 in the same layer of MNG 17001 (star). r = regurgitalite, c = conchostracan, a = arthropod, b = bone, m=´*Megatambichnus*´. (**F**) MNG 17001 before preparation, bottom view. (**G**) MNG 17001 after preparation, bottom view. The arrow indicates the direction of the North compared to the specimen in situ. Scale bars (F-G): 1 cm.

MNG 17001 was found in the stratigraphic interval named BRO II, between the tabular sandstone at the base of the Bromacker succession (BRO III) and the cross-stratified sandstone package in the upper part of the succession (BRO I) (Fig. 1). The interval BRO II is characterised by tabular laminated mudstone (BRO II A, B, E) and cross-stratified to tabular, fine-grained sandstone (BRO II C and D), erosive on the underlying layers and thinning-upwards. MNG 17001 comes from the topmost part of BRO II C in sector WE3 of the western excavation site (Fig. 1). This is a thin (about 5 cm) fine-grained, tabular sandstone layer with a few mudstone lenses. In the same horizons, other fossils were found, including conchostracans, arthropods, isolated bones and scratch traces informally named “*Megatambichnus*”^16^. BRO II can be interpreted as floodplain deposition, which could be overbank (A, B, E) or potentially crevasse splay (C, D). The flow energy was relatively low at the finding spot, as testified by the tabular layers and by the occurrence of mudstone lenses, conchostracans and arthropod body fossils. No burrowing/digging structure was directly associated with MNG 17001.

## Results

### Skeletal content and systematic assignment

**Fig. 2:**
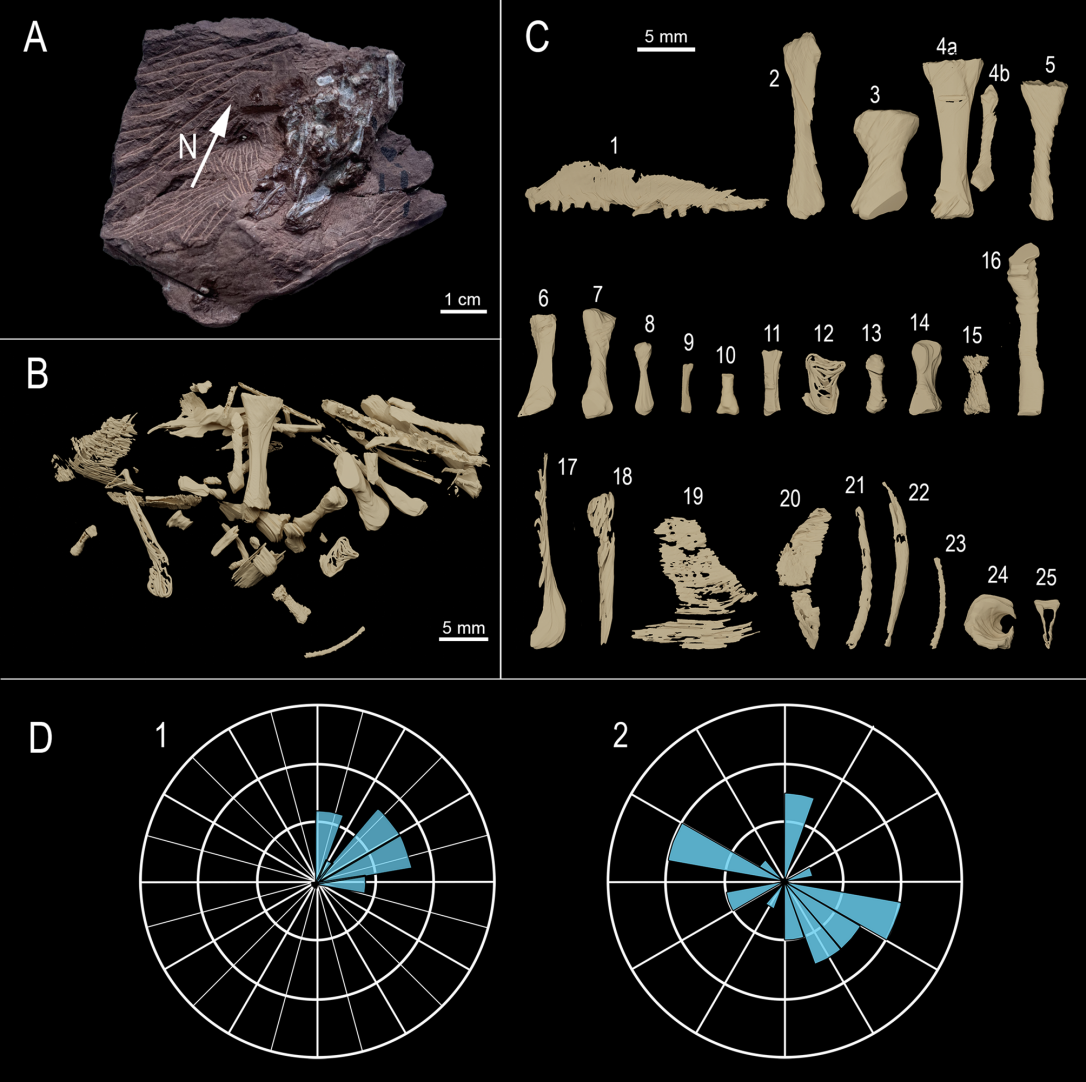
MNG 17001: complete specimen in bottom view (**A**), segmented content of the entire cluster in top view (**B**), and close up view on the identifiable bone remains (**C**). measurements, morphological descriptions and taxonomic assignments are provided in SI. Rose diagrams depicting the skeletal remain orientation (**D**) in 360° (D_1_) and 180° (D_2_), where opposite orientations are treated as equivalent axes.

MNG 17001 consists of a compact cluster including 41 small (< 20 mm long) disarticulated bones. The cluster measures approximately 5.2 cm in length, 3.1 cm in width, and 1.4 cm in thickness. The specimen was µCT-scanned and 41 distinct bones could be segmented out of the matrix (Fig. 2A, B and MovieS1 and S2). Additionally, few very fragmentary bone remains were retrieved. A Rayleigh test was performed, rejecting the null hypothesis of uniformity in a circular distribution (*p*-value < 0.05) indicating that the skeletal remains are not randomly oriented but show a preferred alignment (to the North), which supports the interpretation of the bone cluster as a bromalite^9^.

The bones consist mostly of small and complete skeletal elements. This preservation allows for morphological comparison with more articulated specimens known from the Bromacker locality. Based on comparative osteological analysis, the regurgitalite studied here contains a maxilla assigned to *Thuringothyris mahlendorffae*, a humerus attributed to *Eudibamus cursoris* and a metapodial element of a diadectid. Additionally, several amniote phalanges and metapodial elements were identified, as well as a tibia and fibula in sub-articulation and shoulder girdle elements, but not assignable to any specific taxon. Measurements, anatomical descriptions and taxonomical assignments are available in the SI.

### Elemental analysis (micro-XRF)

**Fig. 3:**
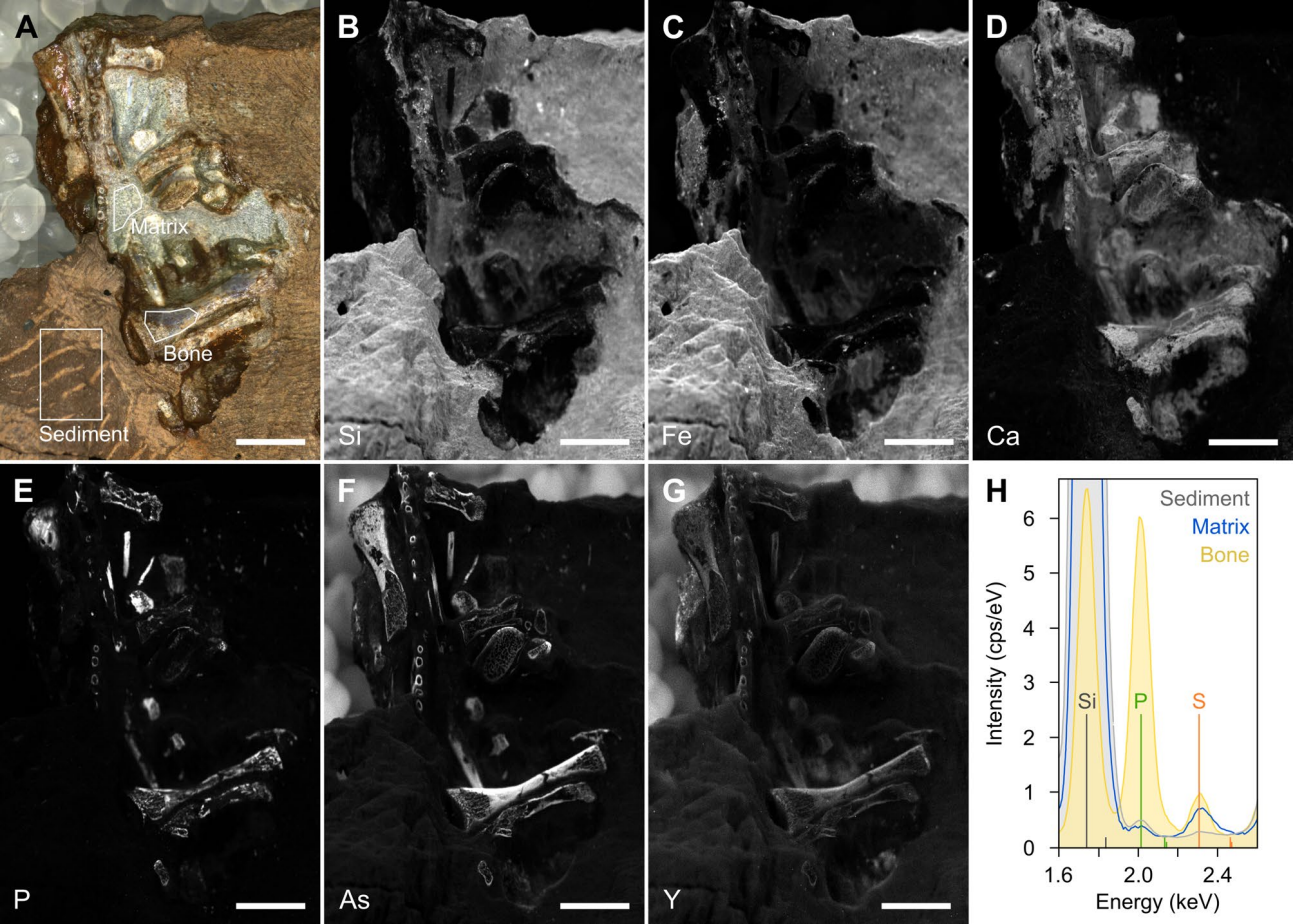
Micro X-ray fluorescence element distribution maps of MNG 17001. (**A**) Optical photograph of the imaged area of MNG 17001. White spherules in the background are PET beads used for non-destructive fixation of the specimen during analysis. (**B-G**) Kα X-ray fluorescence maps of silicon (**B**), iron (**C**), calcium (**D**), phosphorus (**E**), arsenic (**F**), and yttrium (**G**), shown as grayscale images where dark tones denote low concentrations and light tones denote high concentrations. High concentrations of arsenic and yttrium in the PET beads in the background in (**F**) and (**G**) are artefacts. (**H**) Phosphorus and sulphur Kα peaks in X-ray fluorescence spectra of sediment (gray), matrix (blue), and bone (yellow), extracted from the areas in the element distribution map shown in (**A**). Note that the silicon Kα peaks of the sediment and the matrix are truncated and greatly extend the data range shown here. Spectra are normalized by background-matching at ~ 2.5 keV to allow for comparison. Scale bars: 5 mm

Energy dispersive micro X-ray fluorescence (µXRF) was used to gain information on the composition and diagenetic history of MNG 17001. This method has proven to be relevant in several studies focused on regurgitalite identification. These studies show a typical lack of phosphorus content in the matrix directly adjacent to the bone remains whereas in coprolites, the near-bone matrix is usually richer in phosphorus^5,9^. Here, we visualise the distribution of selected major and trace elements in MNG 17001 in element distribution maps (Fig. 3). The resin covering the specimen has been analysed beforehand to ensure that it does not influence the studied spectra. This resin is made of Paraloid B-72 mixed with acetone. X-ray fluorescence spectra and element distribution maps obtained from the area containing the bone fragments (Fig. 3) show high peaks of calcium (Ca), phosphorus (P) in the bones, indicating the expected presence of apatite. In addition, the bones are enriched (Fig. 3 and S2) in arsenic (As), yttrium (Y), and strontium (Sr). The bromalite matrix directly next to the bones reveal peaks of Ca and negligible P, as well as peaks of silicium (Si), aluminium (Al), and iron (Fe), likely reflecting a calcareous cement with clay minerals. As is the case for P, element distribution map further reveals that As, Y, and Sr in the matrix are at similar levels as those in the surrounding sediment. The sediment further away from the bromalite furthermore shows a dominating peak of Si and small peaks of Al, K, and Fe. Hence, the bone is dominated by Ca and P, the matrix directly adjacent to the bone is dominated by Ca and the matrix more distant is neither dominated by Ca nor P.

## Discussion

### Taphonomy

MNG 17001 contains 41 skeletal elements from at least three different taxa compacted in an unusual taphonomic cluster^13^. Such a concentration of remains from different individuals could be caused by an environmental reworking due to attritional processes (abiotic) or digestion of prey by a predator (biotic)^9^. Floodplain-type depositional environments can result in skeletal elements to cluster under the influence of water flow. The bones are tightly packed in a small, isolated, irregular area in the lower part of a thin, tabular, very fine-grained sandstone layer with a clayish base (Fig. 1F). Abiotic disarticulating factors would have caused them to be scattered across the rest of the slab^5^. Although the bone cluster is near a tapering margin of the slab, this location does not justify the bone concentration in a very restricted oval area. Taphonomic artifacts (decay or physical concentrations) may resemble two-dimensional bromalite pellets^1^. However, this interpretation does not apply to MNG 17001 as it is a three-dimensionally preserved cluster. Statistical tests on bone orientations revealed a significant orientation around a specific direction (North). However, due to the lack of sedimentological evidence for transportation, this orientation of the bones is best interpreted as resulting from a biologically reworked cluster, being deposited in a single event and subsequently embedded^3,25^.

MNG 17001 represents the only specimen comprising a multitaxic cluster of small (< 20 mm long) disarticulated bones known from this locality so far. As Bromacker is famous for preserving burrows^21^, skeletal elements could have been concentrated, biotically or abiotically inside such structure^26^. However, the absence of any structure associated to a burrow on the specimen before preparation (Fig. 1F), as well as on the collection spot, is not compatible with this hypothesis^27^. In fact, there is neither identifiable discontinuity on the slab, nor facies change, nor any scratch traces that could be related to a burrow. These diverse taphonomic characteristics therefore do not support an abiotic origin of the bone cluster, but rather suggest a digestive origin (bromalite), likely resulting from the ejection of ingested remains.

### Regurgitalite identification

Skeletal remains of the Bromacker fauna are typically preserved either as fully or partially articulated skeletons, or as isolated elements^13^. Bone clusters similar to MNG 17001 have not been reported since the discovery of the first Bromacker bone in 1974, indicating that these bones were biologically reworked, as suggested by our taphonomic analysis. As stated above, the unusual disposition of the bone assemblage found in MNG 17001 can be identified as a bromalite (fossilised digestive remain). As this bromalite is not preserved inside the body cavity of the producer, it does not represent a consumulite, such as a gastrolite or cololite^1^. This cluster can be associated with digested remains expelled from the producer’s body and could represent either a coprolite or a regurgitalite. Coprolites are much more common in the fossil record^28^. However, MNG 17001 lacks typical coprolitic morphological features such as a clear delimitation from the outer sediments, a fossilised organic matrix^7,9^ embedding the inclusions, and a rather regular shape, for instance spherical, bullet-shaped or (tapered) cylindrical.

In contrast, a combination of several morphological features indicative of a regurgitalite nature (as discussed in refs^1,3,10,11^, is applicable to MNG 17001:

1. MNG 17001 preserves bones that are closely packed and aligned along their longitudinal axis. Such alignment can be observed in bromalites, including regurgitalites, due to the compaction of the prey remains in the digestive tract of the predator^2,9^.
2. MNG 17001 consists primarily of disarticulated bones but includes at least one pair of elements in anatomical alignment: sub-articulated zeugopodials, best interpreted as tibia and fibula (Fig. 2C_4_). Additionally, the presence of complete, elongated limb bones—alongside partially articulated elements—suggests that the remains were only partially digested, supporting interpretation of the specimen as a regurgitalite^10^.
3. MNG 17001 has an irregular overall shape, lacking a clear delimitation from the sedimentary matrix, and it is isolated on the slab lower surface.
4. MNG 17001 and its preserved bones form a cluster and are exposed at the surface, lacking a delimited organic embedding matrix (groundmass); this is typical of regurgitalites^9,28^.

To supplement the morphological observations, we performed a µXRF analysis. The relevance of this method lies in the different elemental composition of coprolites and regurgitalites^5,9,25,29,30^. Due to the digestion of phosphatic elements such as bones^31^, lipids^29^ and other soft tissue of a prey^32^, most of the phosphate will be excreted in faeces^29^. Consequently, the embedding matrix of predator faeces and coprolites has an increased concentration of phosphate^30,32^. In contrast, regurgitalites typically have a low-phosphatic surrounding matrix, most likely due to a shorter digestion period and lacking organic embedding matrix^5,9^.

Our µXRF analysis reveals that the bones exhibit the highest concentrations of phosphorus (P), whereas both the bromalite groundmass and the surrounding host sediment show comparatively low phosphorus concentrations. In typical coprolites, the groundmass is expected to contain much higher levels of phosphorus, which would clearly be distinguishable from the matrix sampled farther from the bone cluster^5^. In addition, the slight enrichment of the bones, but not the groundmass, by yttrium and strontium suggests limited alteration during digestion, consistent with a short digestion period. Yttrium and strontium substitute calcium in calcium phosphates^33^ and can, thus, be expected to show a similar behaviour during digestion. As a result of the combined µCT-based morphological and µXRF-based chemical analyses, MNG 17001 is therefore identified as a regurgitalite as it shows all the diagnostic characteristics of this specific bromalite type^1,4,5,8,9^.

### Stratigraphic significance

**Fig. 4:**
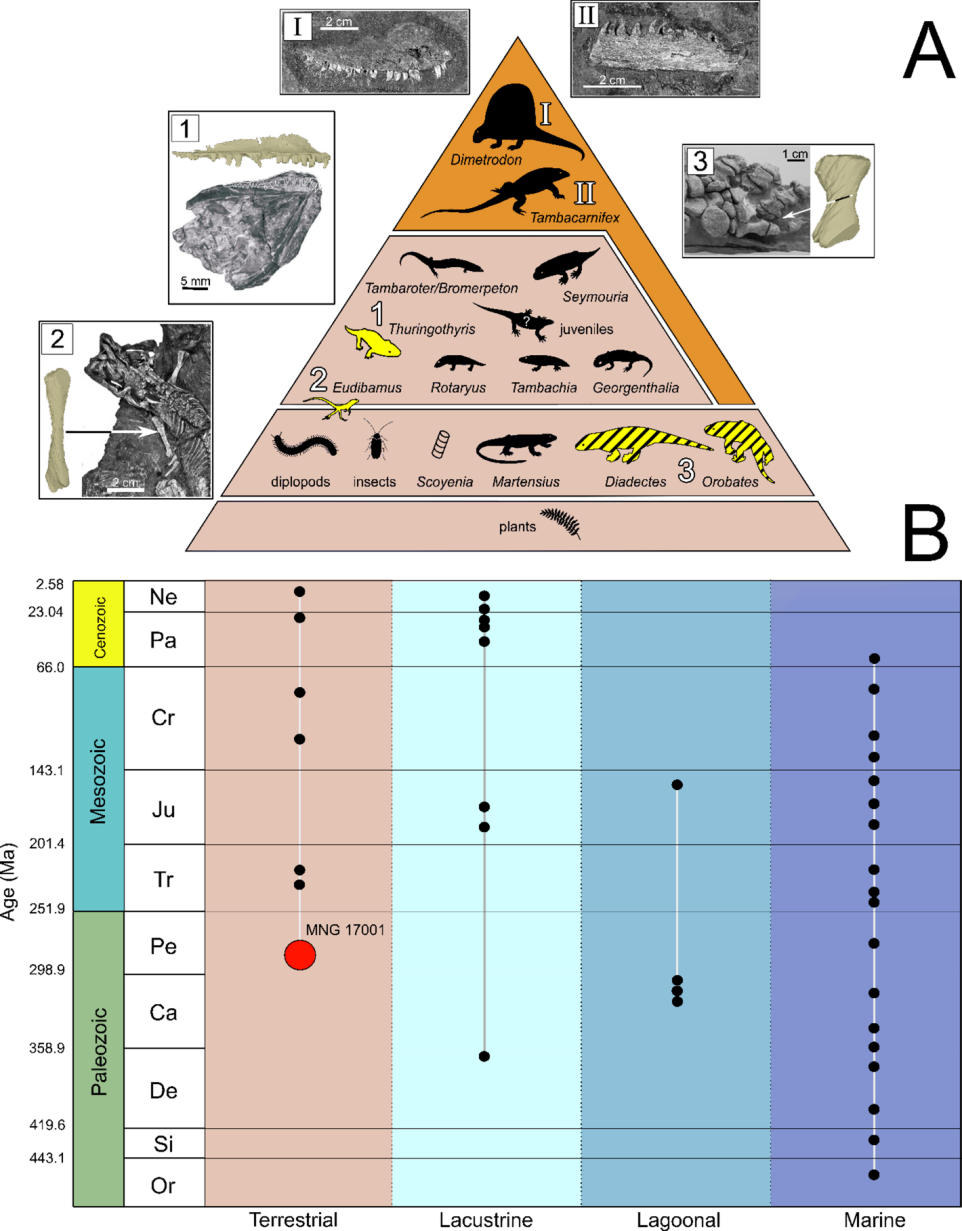
Trophic pyramid (**A**). (modified from Werneburg and Schneider, 2024, Chap. 18^15^) showing the potential producers of the regurgitalite (A_I_: *Dimetrodon teutonis*, A_II_: *Tambacarnifex unguifalcatus*), the taxa identified to species level (coloured in yellow) and family level (yellow stripes) from the Bromacker known fauna, recovered from MNG 17001, along with comparisons between segmented inclusions from the regurgitalite and corresponding more complete skeletal material (A_1_: *Thuringothyris mahlendorffae*; A_2_: *Eudibamus cursoris* A_3_: *Diadectes absitus* or *Orobates pabsti*). Chronostratigraphic distribution (**B**) of all published regurgitalites (each black dot represents a locality in which a regurgitalite is mentioned and/or described) to date, categorised by depositional settings. The figure highlights MNG 17001 (the red dot) as the geologically oldest documented regurgitalite from a terrestrial environment.

**Fig. 5:**
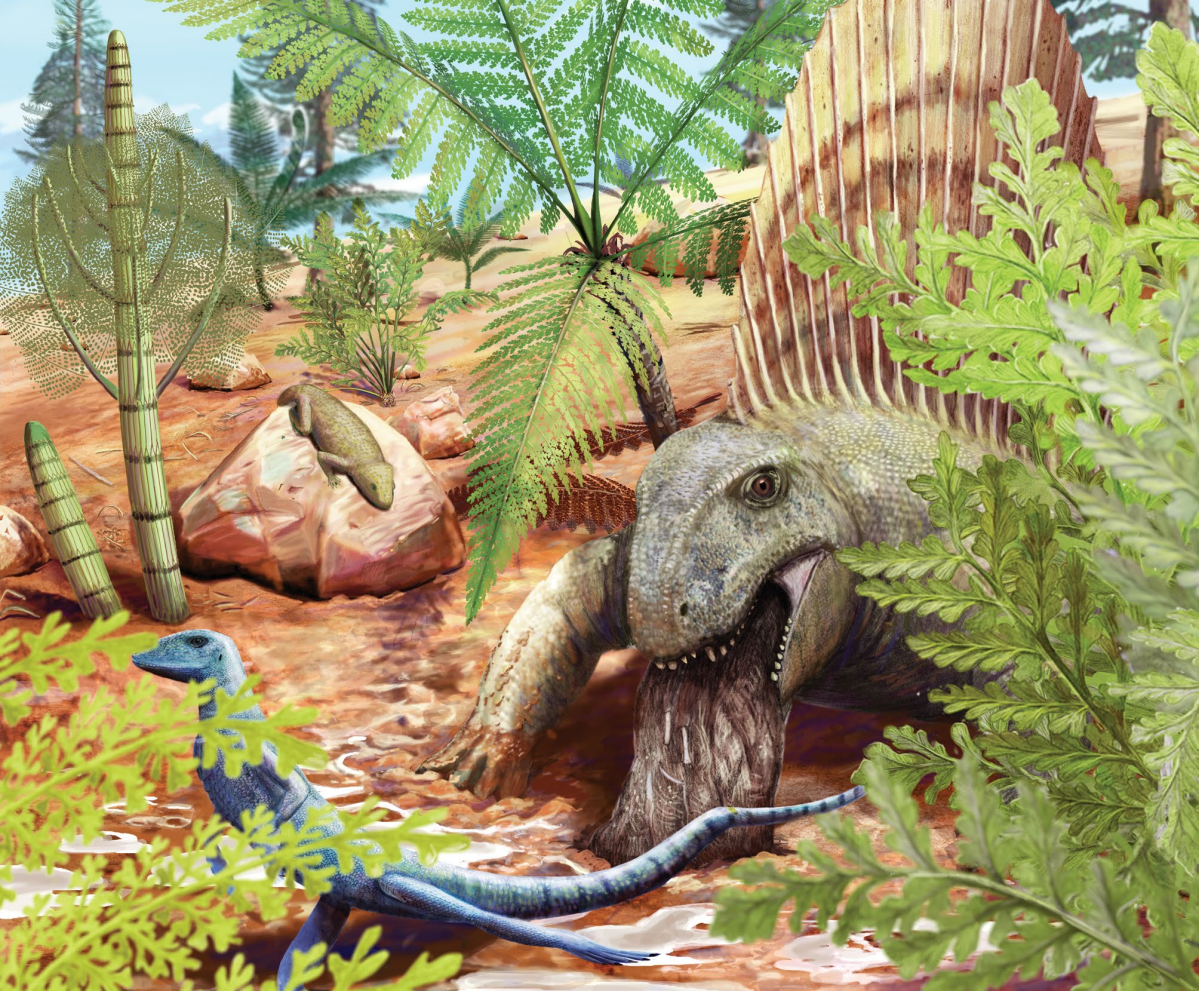
Paleoart by Sophie Fernandez, depicting a specimen of *Dimetrodon teutonis* regurgitating undigestible remains. This artwork also illustrates *Eudibamus cursoris* in the foreground (left), and *Thuringothyris mahlendorffae* in the background on the rock. Floral assemblage reconstructions include *Arnhardtia scheibei*, *Calamites gigas*, *Sphenopteridium germanicum*, and *Walchia piniformis*.

MNG 17001 represents a fossilised regurgitalite from a terrestrial depositional environment, dated to the Sakmarian (ca. 293–290 Ma)^23,24^. Regurgitalites are rare in the Palaeozoic and were, until now, only recovered from marine and transitional depositional environments (Fig. 4). Our specimen represents the geologically oldest tetrapod regurgitalite found in a terrestrial environment, as the Bromacker locality is interpreted as a fluvial to floodplain palaeoenvironment^13^. This discovery suggests that regurgitalites might be found in similar palaeoenvironments from the early Permian. Furthermore, it demonstrates that fully terrestrial carnivorous tetrapods had the physiological ability to regurgitate as early as the Sakmarian stage. The scientific potential and relevance of bromalites has already been emphasized in the recent literature^1,2,5,9,10^. Out of all different types of bromalites (e.g. coprolites, consumulites), regurgitalites are the least likely to be recognised in the field, and hence, are the least studied bromalite^1,2^. This underrepresentation is even more evident for Palaeozoic studies on bromalites, which mainly include marine invertebrate remains^10,34^ and few vertebrates^1,28^. To date, only few convincing examples of pre-Cenozoic regurgitalites have thus far been reported^1,9,11^. Hence, this new regurgitalite from the Bromacker locality holds broad scientific significance, as it provides unprecedented evidence of trophic structure and feeding behaviour within an early Permian terrestrial ecosystem (Fig. 5).

### Physiological, ecological, and trophic insights

The producer of this bromalite was physiologically capable of regurgitation, a common trait in most tetrapods, with exceptions being rodents, lagomorphs and equids^35–37^. Such mechanism allow the predator to regurgitate pre-digested food to feed their offspring, eject parasites and noxious or undigestible material that could become mechanically dangerous within the predator’s body cavity^10,34,38^. For instance, varanid lizards are known to swallow their prey whole, if their size allows it, or to tear large prey chunks. They swallow all the pieces, including bones which are typically orally egested along with other undigestible materials such as hair, feathers, and scales^4,39^. Most of the bones contained in the Bromacker regurgitalite (MNG 17001) are not fractured but largely complete and only superficially digested, which implies that the prey items were swallowed entirely or partly with limited biting or crushing action.

Bromalites also help to elucidate ancient trophic structures, as they are key fossils to track direct trophic interactions^40^. MNG 17001 allows us to identify an important node in the Bromacker trophic network, as three animals of different sizes are preserved in it, suggesting an opportunistic feeding behaviour, meaning the producer likely had a flexible diet, exploiting any faunal resource available, independent of size (small, medium and large tetrapods) and possibly including carrions. Since oral ejection implies a relatively short retention period within the predator’s body cavity, a combination of predation and scavenging, a well-documented behaviour among opportunistic apex predators^4,41,42^, could possibly account for the ingestion of multiple individuals within a limited timeframe. The dominance of large herbivorous vertebrates at the Bromacker locality^12^ suggests a substantial biomass of herbivores dying, thereby providing abundant scavenging opportunities^43^. This idea is consistent with the finding of isolated bones in the same sedimentary layer as MNG 17001 (Fig. 1). Modern opportunistic predators, such as the lace monitor *Varanus varius*, have been observed to regurgitate multiple animals, for instance “four fox cubs, three nestling rabbits and three large blue-tongued skinks”^44^. The low number of bones assigned to each taxon (such as the diadectid metapodial element, see Fig. 2C_3_) can also be the result of leftovers from older meals, still retained inside the producer’s stomach. Also, most of the skeletal remains appear to be long bones, representing the least digestible elements, most likely due to dense cortical bone, harder to fully digest.

However, as is the case for all excreted bromalites (unlike consumulites), it is generally challenging to associate the excreted material with its producer. The vertebrate faunal composition of the Bromacker ecosystem is well known and documents an abundance and high diversity of herbivores compared to carnivores^12^. Based on the overall size of the regurgitalite, as well as the size of the fully preserved long bones and comparative data of carnivorous species from the site (Table S3), we hypothesise that only two taxa currently known from the Bromacker locality would have been sufficiently large to prey on the three animals embedded in this regurgitalite. These candidates are the sphenacodontid *Dimetrodon teutonis* and the varanopid *Tambacarnifex unguifalcatus*^12^. Their respective snout-vent length was estimated at 55 cm for *Dimetrodon teutonis* and 50 cm for *Tambacarnifex unguifalcatus* by Berman et al. 2023^12^. Our estimations based on new measurements and compared with closely related taxa (see Methods) indicate that their snout-vent length could reach up to 80 cm for *Dimetrodon teutonis* and 73 cm for *Tambacarnifex unguifalcatus.* Despite their relatively small size (snout-vent length inferior than one meter long, see Table S3), these two taxa are unequivocally the two largest animals from the Bromacker paleofauna (see Table S3). In fact, the Bromacker skeletal fauna as well as the ichnofauna were characterised exclusively by relatively small animals, different from other Euramerican correlatives^12^, and this is probably not a taphonomic or research bias, because of the large abundance and diversity of both the skeletal and ichnological records^19^. Furthermore, both *Dimetrodon teutonis* and *Tambacarnifex unguifalcatus* lack serrated teeth^45,46^ — a feature typically associated with tearing flesh and bones^47^. The absence of serrations is consistent with our hypothesized feeding behaviour: swallowing prey whole or at least in large parts. However, due to the scarcity of material, we currently cannot state with certainty how these two apex predators differed from each other in the trophic guild and in feeding behaviour^48^. They are both recognised as apex level in the trophic chain^12,48^.

The third largest and possibly true carnivorous taxon is *Seymouria sanjuanensis*^48^. With a maximum snout-vent length of 36.5 cm (see Table S3) *Seymouria* is considerably smaller than *Dimetrodon teutonis* and *Tambacarnifex unguifalcatus.* However, its dentition indicates that it was likely able to ingest insects and small to medium sized tetrapods such as *Thuringothyris mahlendorffae* and *Eudibamus cursoris.* Nonetheless, based on its maximum skull dimension it is highly unlikely that it could have ingested large portions of the much larger and more heavily built diadectids. The other possibly true carnivorous taxa, the trematopid temnospondyls *Rotaryus gothae* and *Tambachia trogallas*, are considered too small (see Table S3) for ingesting the preserved prey animals and producing the regurgitalite.

Considering all evidence, the apex predators *Dimetrodon teutonis* and *Tambacarnifex unguifalcatus* represent the mostly likely producers of the Bromacker regurgitalite (MNG 17001). In the future, additional material from the Bromacker carnivores, but especially *Dimetrodon teutonis* and *Tambacarnifex unguifalcatus*, will potentially allow to better model the gape of each predator and more quantitatively assess and differentiate the feeding behaviour of these predators. The multitaxic composition of MNG 17001 indicates an opportunistic feeding behaviour of its producer. Many modern apex predators, such as *Varanus komodoensis*^4^, *Crocodylus porosus*^42^ *and Panthera leo*^49^, are known to adopt an opportunistic feeding strategy including scavenging. This shows that apex predators at the Bromacker locality did not exclusively prey on large and medium-sized herbivores, but also on smaller individuals, providing novel insights into the feeding behaviour of apex predators in late Palaeozoic terrestrial ecosystems.

## Materials and methods

### Specimen

The specimen MNG 17001 is stored at the Friedenstein Stiftung, Gotha, Thuringia, Germany. It was found during the excavation 2021 at the Bromacker locality (Tambach Formation, Germany). UTM coordinates of the finding spot are: 614053.8672; 5629923.155; 436.1273. The specimen was prepared at the Museum für Naturkunde Berlin, Germany and analysed first-hand and photographed at the same institution, using a Canon EOS 90D digital camera.

### µCT scan and segmentation

MNG 17001 was scanned using the x-ray computed tomography equipment (Yxlon FF85 CT) at the Museum für Naturkunde Berlin. Scan parameters were set to 160 kV voltage and 140 µA current with 3000 images per 360° at an unspecified exposure time and an effective voxel size of 0.020 mm. The scan was performed using a cone beam setup with a circular trajectory, a focus-to-object distance (FOD) of 666.67 mm, and a focus-to-detector distance (FDD) of 5000 mm. The detector, a Perkin Elmer Y.Panel 4343 CT, captured 16-bit projections with a resolution of 2850 × 2860 pixels and a pixel size of 0.15 mm. Cone beam reconstruction was performed using Yxlon CeraRecon (version 6.1.1) with a voxel size of 0.020 mm³ and a volume size of 2850 × 2850 × 2860 voxels. Beam hardening correction was applied using a single-material model (Steel_X10CrMoVNb) with a copper filter of 0.1 mm thickness. Auto-alignment optimization was enabled, and truncation correction was applied, though metal artefact reduction and scatter correction were not used. Noise reduction was performed with spatial and range sigma values of 2 and 1.5, respectively. Elements were visualized and digitally segmented in AmiraZIBEdition 2024.04. The 3D models resulting from the segmentation were outputted to stereolithography file format (*.stl) and imported into Blender 4.3 (https://www.blender.org*).*

### Elemental analysis

The elemental composition of MNG 17001 was analysed entirely non-destructively using a Bruker M4 Tornado Plus energy dispersive micro X-ray fluorescence (µXRF) spectrometer at the Museum für Naturkunde Berlin. An element distribution map of the bone cluster was obtained using a 30-W Rh metal-ceramic micro-focus tube with a Be window coupled to a polycapillary lens. The spot size of the X-ray beam on the sample surface was approximately 20 μm (calibrated for Mo-Kα radiation) and maximum energy settings of 50 kV acceleration voltage and 600 µA beam current were used for obtaining the element distribution map. The pixel size and dwell time per pixel was set to 20 μm and 40 ms, respectively, and the total map area was 1160 × 1665 px or 23.2 × 33.3 mm. To improve the depth of field of the element map, an aperture of 1000 μm, incorporated in the patented Aperture Management System of the µXRF instrument, was used. In order to avoid attenuation of fluorescence X-rays by air molecules in the sample chamber as much as possible, the map was acquired at a sample chamber pressure of 2 mbar. Characteristic fluorescence X-rays were detected simultaneously by two 60-mm^2^ silicon drift detectors with energy resolutions of ≤ 145 eV (Full Width at Half Maximum calibrated for Mn-Kα radiation). Deconvolution and quantification of the acquired energy-dispersive X-ray spectra and background subtraction was done using the Bruker M4 Version 1.6 operating software. A Fundamental Parameters algorithm^50,51^ that is based on the Sherman Eq. 5^2^ and implemented in the operating system was used to obtain elemental concentrations on the basis of net counts for each element of interest. Data visualization was done using the Bruker M4 Version 1.6 operating software.

### Statistics

Expelled bromalites typically show an alignment of their inclusions along their long axis. To test whether the skeletal remains of MNG 17001 show a significant preferred orientation, we performed statistical tests. The analyses and visualisations were performed in Rstudio (Version: 2024.12.1 + 563), using a Rayleigh Test of Circular Uniformity^53–55^ and a Watson’s Two-sampled-Test of Homogeneity^53^.

### Measurement and sizes estimations

Measurements of skeletal elements from the Bromacker fauna were taken by Lorenzo Marchetti and Aurore Canoville with a calliper, in the frame of a separate project. Due to missing skeletal material, snout-vent length, skull length and skull width of *Dimetrodon teutonis* and *Tambacarnifex unguifalcatus* had to be roughly estimated using skeletal material from phylogenetically close taxa. For *Dimetrodon teutonis*, the humerus and femur length were measured and compared with the holotype of *Dimetrodon milleri.* For *Tambacarnifex unguifalcatus*, the humerus and femur length were measured and compared with *Aerosaurus wellesi*, a closely related member of Varanodontinae. A rule of three was used, based on humerus and femur length, to estimate snout-vent length and skull measurements.

## Supplementary Information

Below is the link to the electronic supplementary material.

Supplementary Material 1

Supplementary Material 2

Supplementary Material 3

## Data Availability

The data reported in this paper are detailed in the main text and in the Supplementary Information. The raw CT data used can be accessed at MorphoSource: https://www.morphosource.org/projects/000776952/temporary_link/UUCxeysjyaoTEyPAHyDTQgYN? loc ale=en.
