# Supplementary material for: Early Permian terrestrial apex predator regurgitalite indicates opportunistic feeding behaviour
Source: Sci Rep. 2026 Jan 30;16:1087. doi: 10.1038/s41598-025-33381-0 (PMC12859032; doi:10.1038/s41598-025-33381-0)
Supplement: Supplementary file 1 — Supplementary Material 1 [file 41598_2025_33381_MOESM1_ESM.pdf]

Supplementary information for

Early Permian terrestrial vertebrate apex predator regurgitalite indicates opportunistic feeding behaviour

Arnaud Rebillard*, Andréas Jannel, Lorenzo Marchetti, Mark J. MacDougall, Christopher Hamann, Jean-Sébastien Steyer, Jörg Fröbisch

*Arnaud Rebillard

Email: Arnaud.Rebillard@mfn.berlin

This PDF file includes:

Supporting text

Figures S1 to S2

Tables S1 to S2

Legends for Movies S1 to S2

SI References

Other supporting materials for this manuscript include the following:

Movies S1 to S2

Supporting Information Text

Description of the identifiable skeletal remains from MNG 17001:

C_1: A left maxilla (Figure 2C_1), 20,9 mm long and 3 mm high, preserving ten small, recurved sub-conical monocuspid teeth. Its dorsal part consists of a low blade, the outline of which is slightly irregular, indented and hence partially eroded, possibly from partial digestion. The tooth row is relatively straight anteroposteriorly and bears seemingly homodont dentition. The maxilla preserves ten teeth, but space for at least 17-18 tooth positions. However, it is unclear whether the maxilla is preserved entirely or incomplete anteriorly and posteriorly, allowing an even higher tooth count. The tooth crown apex is mostly not preserved, except for the preserved tooth position 1, with a height of 1,20 mm and indicating a slightly recurved morphology. The first tooth appears slightly smaller than the following five preserved teeth, whereas the posterior teeth seem to decrease again in size posteriorly. The tooth implantation is pleurodont. Their inner structure, detectable in apical view, does not present any plicidentine. Based on the shape and size of this bone as well as the tooth morphology, we refer this maxilla to *Thuringothyris mahlendorffae.*

C_2: An elongated, slender bone with only slightly expanded proximal and distal epiphyses that are twisted along the diaphysis with an angle of about 30° (Figure 2C_2) corresponding to the humerus of a small but slender reptile. Based on the relative proportions of known humeri from the Bromacker locality, the element best fits the bolosaurid *Eudibamus cursoris*.

C_3: A short, robust bone with broadened, pronounced epiphyses of similar size and with flat proximal and distal ends and slightly concave medial and lateral sides (Figure 2C_3). Based on the measures, this bone is about 50% more robust than the other preserved remains, and identified here as a diadectid metapodial V. In comparison, diadectid metapodials I and II are too thick and diadectid metapodial III and IV are too elongated and with a more marked medial and lateral concavity. It differs from synapsid phalanges because of the similar size and morphology of the proximal and distal ends, the moderate lateral and medial concavity and the absence of strongly anterodorsally inclined proximal and distal surfaces.

C_4: An elongated, straight and somewhat robust bone (Figure 2C_{4a}) with a straight and broad proximal end and a slightly less broad but equally straight distal end, most likely corresponding to a tibia which appears to have been preserved in sub-articulation with another elongated bone (Figure 2C_{4b}) that is much less complete and only about have the width of the tibia, and best resembles a fibula.

C_5: An elongated, straight bone (Figure 2C_5) with a straight and broad proximal end and a narrower, rounded distal end. This bone is identified as an amniote metapodial element.

C_6: An elongated, straight and somewhat robust bone (Figure 2C_6) with a straight and broad proximal end and a narrower, rounded distal end which appears flattened, likely damaged in the digestion/feeding processes.

C_7: An elongated, straight bone (Figure 2C_7) with a rounded proximal end and a similarly broad, rounded distal end. This bone is identified as an amniote metapodial element.

C_8: A small elongated, straight bone (Figure 2C_8) with a rounded proximal end and a similarly broad, rounded distal end. This bone is identified as an amniote metapodial element.

C_9: A small (<5 mm) elongated, straight bone (Figure 2C_9) poorly preserved with a slightly rounded proximal end and straight distal end. This bone is identified as an amniote metapodial element or phalanx.

C_{10}: A small (<5 mm) broken part from a small elongated bone (Figure 2C_{10}) with a straight, narrow shaft, and slightly expanded proximal end with a straight articulation facet. The distal epiphysis is missing. This bone is identified as an amniote metapodial element or phalanx.

C_{11}: An elongated, straight bone (Figure 2C_{11}) with a rounded, flattened, slightly convex proximal end and rounded, slightly concave, distal end. This bone is identified as an amniote metapodial element or phalanx.

C_{12}: A broad, straight bone (Figure 2C_{12}) with a rounded, flattened, slightly convex proximal end and rounded, slightly concave, distal end. This bone is identified as a tetrapod metapodial element or phalanx.

C_{13}: A elongated, straight bone (Figure 2C_{13}) with a convex proximal end and concave distal end. This bone is identified as an amniote metapodial element or phalanx.

C_{14}: A elongated, straight bone (Figure 2C_{14}) with a convex proximal end and concave distal end. This bone is identified as an amniote phalanx.

C_{15}: A small (<5 mm) elongated, straight bone (Figure 2C_{15}) with a convex proximal end and concave distal end. This bone is identified as an amniote phalanx.

C_{16}: A elongated, slender and straight bone (Figure 2C_{16}) with damaged ends. This bone is identified as an amniote zeugopodial element.

C_{17}: A poorly preserved elongated, straight bone (Figure 2C_{17}) with only one preserved rounded epiphysis. This bone is identified as an amniote fragmentary long bone.

C_{18}: A poorly preserved elongated, straight bone (Figure 2C_{18}). Only one rounded end is preserved. This bone is identified as a tetrapod fragmentary long bone.

C_{19}: A poorly preserved flat, plate-like element (Figure 2C_{19}). This bone is tentatively identified as a tetrapod scapulocoracoid.

C_{20}: A flat, broad and strongly curved bone (Figure 2C_{20}). This bone is identified as a poterntial tetrapod clavicle.

C_{21}: A thin, very elongated curved bone (Figure 2C_{21}). This bone is identified as a tetrapod rib.

C_{22}: A thin, very elongated curved bone (Figure 2C_{22}). This bone is identified as a tetrapod rib.

C_{23}: A thin, very elongated curved bone (Figure 2C_{23}). This bone is identified as a tetrapod rib.

C_{24}: A small (<5 mm) damaged rounded and thick bone (Figure 2C_{24}). This bone is identified as a partial amphicoelous vertebral pleurocentrum, likely of an amniote.

C_{25}: A small (<5 mm) bone shaped like a thick-walled, elongated isosceles triangle (Figure 2C_{25}), enclosing an empty space. The proximal end is thickest and slightly convex. This bone is identified as a tetrapod haemal arch.

Figures

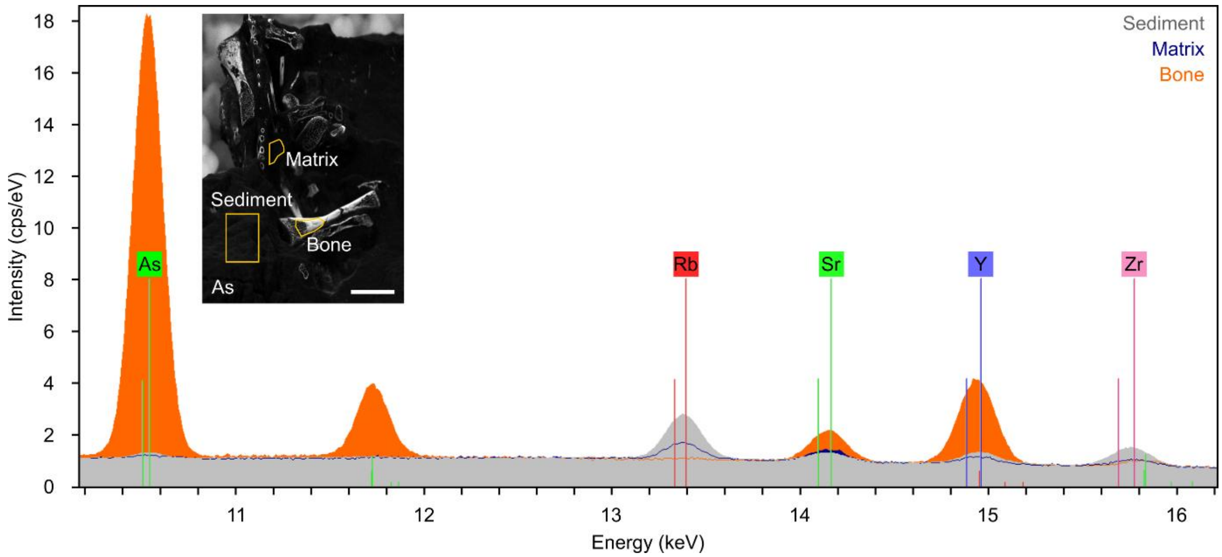

FigureS1: Arsenic, rubidium, strontium, yttrium, and zirconium Kα peaks in X-ray fluorescence spectra of representative areas of sediment (gray), bromalite matrix (blue), and bone (orange) materials. Data were extracted from the areas in the element distribution map shown in the inset in the upper left. Spectra are normalized by background-matching in the range of 12.2–13.0 keV to allow for comparison. Note that the bone is enriched in arsenic, strontium, and yttrium and depleted in rubidium compared to both the bromalite matrix and surrounding sediment.

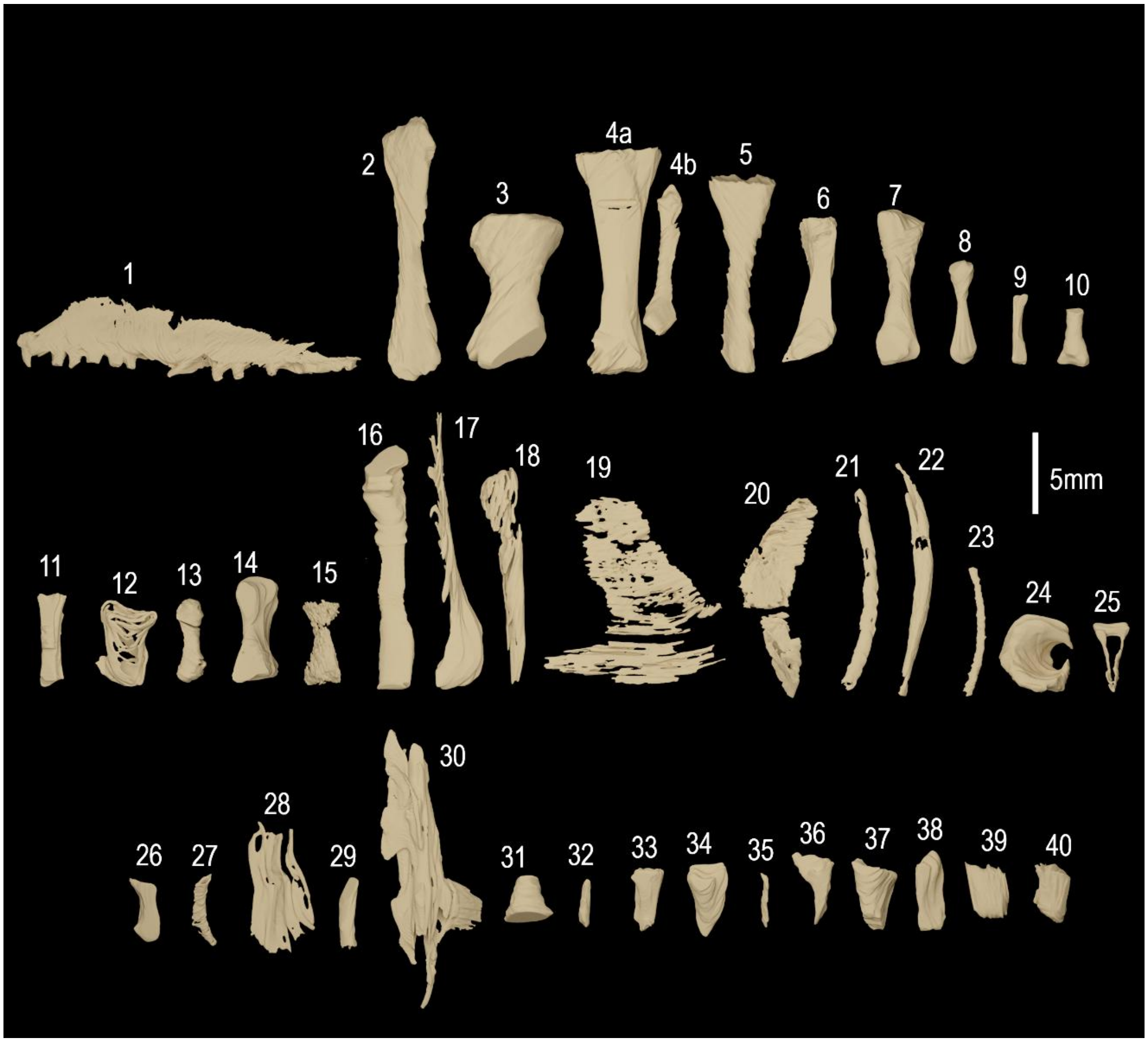

FigureS2: All 41 bone remains retrieved from the regurgitalite. Measurements, morphological descriptions, anatomical and taxonomical assignment for each remain are detailed in the Supporting Information Text and TableS1.

Tables

TableS1: List of measurements of long bones preserved in the regurgitalite, associated with their anatomical and taxonomical assignment, when possible. Measurements include the bone length (L), minimum width (W_{min}), maximum width (W_{max}), minimum diameter (D_{min}), maximum diameter (D_{max}) and average diameter.

**Number**	**Length (in mm)**	**Width extremity (in mm)**	**Width mid-shaft (in mm)**	**Longest diameter**	**Shortest diameter**	**Anatomical identification**	**Taxonomical affiliation**
1	20.88					maxilla	*Thuringothyris mahlendorffae*
2	15.73	3.312	1.38	1.62	1.28	humerus	*Eudibamus cursoris*
3	9.48	5.59	2.97	3.05	2.63	Metapodial V	diadectid
4a	13.66	5.106	2.208	2.26	1.56	tibia	amniote
4b	9.80	1.794	1.104	1.14	0.62	fibula	amniote
5	11.73	4.002	1.518	1.42	1.04	metapodial element	amniote
6	8.69	2.622	1.518	1.78	1.23	metapodial element	amniote
7	9.25	3.174	1.242	1.35	1.18	metapodial element	amniote
8	6.30	1.7	0.63	0.66	0.62	metapodial element	amniote
9	4.19					metapodial element/ phalanx	tetrapod

10	3.83					metapodial element/ phalanx	amniote
11	6.43					metapodial element/ phalanx	amniote
12	5.98					metapodial element/ phalanx	tetrapod
13	5.54					metapodial element/ phalanx	amniote
14	6.43	3.01	1.7952	1.64	1.52	phalanx	amniote
15	4.97	2.484	1.104	1.37	0.57	phalanx	amniote
16	14.63					zeugopodial element	amniote
17	16.42	3.45				fragmentary long bone	amniote
18	12.97	3.036	1.794			fragmentary long bone	tetrapod
19	11.75					scapulocoracoid	tetrapod
20	12.12					clavicle	tetrapod
21	11.87					rib	tetrapod
22	14.35					rib	tetrapod
23	8.68					rib	tetrapod

24	4.64					pleurocrentrum	amniote
25	4.28					haemal arch	tetrapod
26	4.34					bone fragment	tetrapod
27	5.24					bone fragment	tetrapod
28	9.42					bone fragment	tetrapod
29	5.09					bone fragment	tetrapod
30	19.30					bone fragment	tetrapod
31	2.84					bone fragment	tetrapod
32	3.25					bone fragment	tetrapod
33	4.34					bone fragment	tetrapod
34	4.94					bone fragment	tetrapod
35	4.30					bone fragment	tetrapod
36	3.94					bone fragment	tetrapod
37	4.64					bone fragment	tetrapod

38	2.70					bone fragment	tetrapod
39	4.04					bone fragment	tetrapod
40	3.36					bone fragment	tetrapod

TableS2: List of all reported regurgitalites, sorted by age from the oldest (Late Ordovician) to most recent (Pliocene). This data forms the basis for Figure 4 in the main text of the manuscript.

Age	Locality	Environment	Reference
Late Ordovician	Soom Shale Lagerstätte (South Africa)	marine	Aldridge et al. (2006)[1]
Silurian	Holy Cross Mountains (Poland)	marine	Brachaniec et al. (2016)[2]
Early Devonian	Shales of Forfarshire / Duntrune Quarry (Scotland)	marine	Burrow and Turner (2010)[3]
latest Devonian	Hangenberg Black Shale of the southern Maïder Basin (Morocco)	marine	Klug and Vallon (2018)[4]
Frasnian	Dębnik (Poland)	marine	Salamon et al. (2014)[5]
Fammenian	Strud quarry (Belgium)	lacustrine	Robin et al. (2022)[6]
Mississippian	Mokrá Quarry (Czech Republic) / Ostrówka, Orlej, Czatkowice (Poland)	marine	Salamon et al. (2014)[5]
Mississippian	Bear Gulch Lagerstätte (Montana, USA)	marine	Hunt et al. (2012)[7]
Pennsylvanian	Kinney Brick Quarry and Tinajas Lagerstätten (New Mexico, USA)	lagoonal	Hunt et al. (2012)
Early Pennsylvanian	Carbondale Formation (Indiana, USA)	marine	Zangerl and Richardson (1963)[8]
Pennsylvanian	Mazon Creek	lagoonal	
Pennsylvanian	Puertollano basin (Spain)	lagoonal	Soler-Gijón, R., & Ruiz, A. D. (2023)[9]
Early Permian	Bromacker (Germany)	terrestrial	Rebillard et al. xxxx
Early Permian	Mangrullo Formation (Uruguay)	marine	Silva et al (2017)[10]
Early Triassic	Longtan section (China)	marine	Yao et al. 2024[11]
Middle Triassic	Langmushan section (China)	marine	Ye et al. (2023)[12]
Middle Triassic	Wojkowice quarry (Poland)	marine	Salamon et al. (2012)[13]

Late Triassic	Polzberg outcrop (Austria)	marine	Lukeneder et al. (2020)[14]
Late Triassic	Dolomia di Forni (Italy)	marine	Dalla Vecchia et al. (1989)[15]
Late Triassic	Owl Rock Member (Arizona, USA)	terrestrial	Gordon et al. (2020)[16]
Lower Jurassic	Posidonia Shale (Germany)	marine	Keller (1977)[17]; Vallon (2012)[18]; Thies and Hauff (2013)[19]; Cooper et al. (2024)[20]
Lower Jurassic	Yuzhou deposit (China)	lacustrine	Ren et al. (2024)[21]
Lower Jurassic	Osteno outcrop (Italy)	marine	Pinna et al. (1985)[22] ;Garassino and Donovan (2000)[23]
Middle Jurassic	Zia Sand Formation (New Mexico, USA)	marine	Zatoń et al. (2007)[24]; Zatoń and Salamon (2008)[25]
Middle Jurassic	Sadowa Góra Quarry (Poland)	marine	Niedźwiedzki et al. (2021)[26]
Middle Jurassic	Biefeld (Germany)	marine	Horstmann and Maier (1957)[27]
Middle Jurassic	Morrison Formation (Utah, USA)	lacustrine	Foster et al. (2022)[28]
Late Jurassic	Nusplingen (Germany)	marine	Grawe-Baumeister et al. (2000)[29] ; Dietl and Schweigert (2001)[30]; Vallon (2012)[18]; Stevens et al. (2014)[31]; Viohl (2015)[32]; Hoffmann et al. (2020)[33]
Late Jurassic	Cismon Creek (Italy)	marine	Serafini et al. (2022)[34]
Late Jurassic	Solnhofen (Germany)	lagoonal	Broili (1938)[35]; Janicke and Schairer (1970)[36]; Janicke (1970)[37]; Wellnhofer (1970)[38]; Barthel (1978)[39]; Bennett (2014)[40]; Witton (2018)[41]; Hoffman et al. (2020)[33]
Early Cretaceous	Las Hoyas (Spain)	lacustrine	Sanz et al. (2001)[42]
Early Cretaceous	Jehol deposit (China)	terrestrial	Zheng et al. (2018)[43]
Early Cretaceous	Xiagou Formation (China)	lacustrine	O'Connor et al. (2025)[44]

Late Cretaceous	Lägerdorf and Kronsmoor (Germany)	marine	Neumann (2000)[45]
Late Cretaceous	Kansas, USA	marine	Bishop (1975)[46] ; Hattin (1996)[47], Everhart (1999, 2003, 2004)[48–50]
Late Cretaceous	En Nammour (Lebanon)	marine	Capasso (2019)[51]
Late Cretaceous	Nammoûra Lagerstätte (Lebanon)	marine	Dalla Vecchia and Chiappe (2002)
Late Cretaceous	Egg Mountain (Montana, USA)	terrestrial	Freimuth et al. (2021)[52]
Paleocene	Punta Peligro locality (Argentina)	marine	Muzzopappa et al. (2021)[53]
Middle Eocene	British columbia (Canada)	lacustrine	Wilson (1977, 1977, 1980, 1987)[54–57]
Middle Eocene	Florissant Formation (Colorado, USA)	lacustrine	Buskirk et al. (2015)[58]
Middle Eocene	Messel (Germany)	lacustrine	(Mayr and Schaal (2016)[59]; Gunnell et al. (2018)[60]; Smith et al. (2018)[61]
Middle Eocene	Omomys Quarry (Wyoming, USA)	lacustrine	Murphey et al. (2001) (62)
Latest Eocene	Chardron Formation (South Dakota, USA)	lacustrine	Hunt and Lucas (2007)[63]
Oligocene	White River Formation (Wyoming, USA)	lacustrine	Lucas et al. (2012)[64]
Late Oligocene	Enspel (Germany)	lacustrine	Engesser and Storch (1999)[65]; Smith and Wuttke (2015)[66]
Late Oligocene	Zia Sand Formation (New Mexico, USA)	terrestrial (fluvial)	Gawne (1975)[67]
Early Miocene	Foieta la Sarra-A (Spain)	lacustrine	Álvarez-Parra et al. (2021)[68]
Upper Miocene	Andalhuala Formation (Argentina)	terrestrial (fluvial)	Nasif et al. (2009)[69]
Pliocene	Verde Formation (Arizona, USA)	lacustrine	Walton (1990)[70], Czaplewski (2011)[71]

Legends for movies

MovieS1: Animation showing MNG 17001 spinning. The matrix surrounding the skeletal elements slowly becomes transparent, allowing to see the bone cluster in three dimensions.

MovieS2: Animation showing the bone remains moving from their original position in the sedimentary matrix and positioning themselves outside of it, in a position close to the way they are displayed in Figure 2

SI References

1. Aldridge, R. J., Gabbott, S. E., Siveter, L. J. & Theron, J. N. Bromalites from the Soom Shale Lagerstätte (Upper Ordovician) of South Africa: palaeoecological and palaeobiological implications. *Palaeontology* **49**, 857–871 (2006).
2. Brachaniec, T., Leko, K. & Wieczorek, A. Regurgitalite from the Silurian of Holy Cross Mountains, southern Poland. *Neues Jahrbuch für Geologie und Paläontologie - Abhandlungen* **280**, 331–334 (2016).
3. Burrow, C. & Turner, S. Reassessment of '*Protodus' scoticus* from the Early Devonian of Scotland. *Morphology, Phylogeny and Paleobiogeography of Fossil Fishes* 123–144 (2010).
4. Klug, C. & Vallon, L. H. Regurgitated ammonoid remains from the latest Devonian of Morocco. *Swiss J Palaeontol* **138**, 87–97 (2018).
5. Salamon, M. A., Gorzelak, P., Niedźwiedzki, R., Trzęsiok, D. & Baumiller, T. K. Trends in shell fragmentation as evidence of mid-Paleozoic changes in marine predation. *Paleobiology* **40**, 14–23 (2014).
6. Robin, N., Noirit, F., Chevrinais, M., Clément, G. & Olive, S. Vertebrate predation in the Late Devonian evidenced by bite traces and regurgitations: implications within an early tetrapod freshwater ecosystem. *Papers in Palaeontology* **8**, e1460 (2022).
7. Hunt, A. & Lucas, S. Classification of vertebrate coprolites and related trace fossils. *New Mexico Museum of Natural History and Science* **Bulletin 57**, 137–146 (2012).
8. Zangerl, R. & Richardson, E. S., Jr. *The Paleoecological History of Two Pennsylvanian Black Shales*. vol. v.4 (1963) (Chicago, Chicago Natural History Museum, 1963, 1963).
9. Soler-Gijón, R. & Díez Ruiz, A. 'Carbonífero de Puertollano' Natural Monument (Puertollano basin, Spain): a window for the knowledge of Early Vertebrates. *Spanish J. Palaeontol.* (2023) doi:10.7203/sjp.26788.

10. Silva, R. R., Ferigolo, J., Bajdek, P. & Piñeiro, G. The feeding habits of Mesosauridae. *Frontiers in Earth Science* **5**, (2017).

11. Yao, M. *et al.* Conodont-bearing bromalites from South China: Evidence for multiple predations on conodonts in the Early Triassic marine ecosystem. *Palaeogeography, Palaeoclimatology, Palaeoecology* **651**, 112377 (2024).

12. Ye, X.-J., Sun, Z.-Y. & Yao, M.-T. A *Keichousaurus*-bearing regurgitalite from the Middle Triassic Xingyi Fauna, Dingxiao of Xingyi City, Guizhou, South China. *Palaeoworld* **33**, 363–373 (2024).

13. Salamon, M. A., Niedźwiedzki, R., Gorzelak, P., Lach, R. & Surmik, D. Bromalites from the Middle Triassic of Poland and the rise of the Mesozoic Marine Revolution. *Palaeogeography, Palaeoclimatology, Palaeoecology* **321–322**, 142–150 (2012).

14. Lukeneder, A. *et al.* Bromalites from the Upper Triassic Polzberg section (Austria); insights into trophic interactions and food chains of the Polzberg palaeobiota. *Scientific Reports* **10**, 20545 (2020).

15. Dalla Vecchia, F., Muscio, G. & R., W. Pterosaur remains in a gastric pellet from Upper Triassic (Norian) of Rio Seazza valley (Udine, Italy). *Gortania - Atti del Museo Friulano di Storia naturale* **10(1988)**, (1989).

16. Gordon, C. M., Roach, B. T., Parker, W. G. & Briggs, D. E. G. Distinguishing regurgitalites and coprolites: a case study using a Triassic bromalite with soft tissue of the pseudosuchian archosaur *Revueltosaurus*. *PALAIOS* **35**, 111–121 (2020).

17. Keller, T. Frassreste im süddeutschen Posidonienschiefer: Jahreshefte Gesellschaft für Naturkunde Württemberg, v. 132. (1977).

18. Vallon, L. H. Digestichnia (Vialov, 1972)–an almost forgotten ethological class for trace fossils. *New Mexico Museum of Natural History and Science, Bulletin* **57**, 131–135 (2012).

19. Thies, D. & Hauff, R. B. A Speiballen from the Lower Jurassic Posidonia Shale of South Germany. *njgpa* **267**, 117–124 (2013).

20. Cooper, S. L., López-Arbarello, A. & Maxwell, E. E. First occurrence of a† coccolepidid fish (? Chondrostei:† Coccolepididae) from the Upper Lias (Toarcian, Early Jurassic) of southern Germany. *Palaeontologia Electronica* **27**, 1–25 (2024).

21. Ren, T.-C., Ma, X.-Y., Wang, Q.-D. & Xu, G.-H. An exceptionally preserved fossil assemblage from the early Jurassic of Chongqing (China) reveals a complex lacustrine ecosystem. *Scientific Reports* **14**, 26147 (2024).

22. Pinna, G., Arduini, P., Pesarini, C. & Teruzzi, G. Some controversial aspects of the morphology and anatomy of *Ostenocaris cypriformis* (Crustacea, Thylacocephala). *Earth and Environmental Science Transactions of the Royal Society of Edinburgh* **76**, 373–379 (1985).

23. Garassino, A. & Donovan, D. T. A new family Of coleoids from the lower Jurassic of Osteno, Northern Italy. *Palaeontology* **43**, 1019–1038 (2000).

24. Zatoń, M., Villier, L. & Salamon, M. A. Signs of predation in the Middle Jurassic of south-central Poland: evidence from echinoderm taphonomy. *Lethaia* **40**, 139–151 (2007).

25. Zatoń, M. & Salamon, M. A. Durophagous predation on middle Jurassic molluscs, as evidenced from shell fragmentation. *Palaeontology* **51**, 63–70 (2008).

26. Niedźwiedzki, R., Surmik, D., Chećko, A. & Salamon, M. A. A regurgitalite of the Middle Triassic (Muschelkalk) from Upper Silesia (Poland). *geol* **47**, 33–40 (2021).

27. Horstmann, G. & Maier, D. Neue Sauriergewölle aus dem Bielefelder Dogger. *Aufschluss* **8**, 17–21 (1957).

28. Foster, J. R., Hunt, A. P. & Kirkland, J. I. Significance of a small regurgitalite containing lissamphibian bones, from the Morrison formation (upper Jurassic), within a diverse plant locality deposit in southeastern Utah, USA. *Palaios* **37**, 433–442 (2022).

29. Grawe-Baumeister, J., Schweigert, G. & Dietl, G. *Echiniden Aus Dem Nusplinger Plattenkalk (Ober-Kimmeridgium, Südwestdeutschland)*. (na, 2000).
30. Dietl, G. & Schweigert, G. *Im Reich Der Meerengel: Der Nusplinger Plattenkalk Und Seine Fossilien*. (F. Pfeil, 2001).
31. Stevens, K., Mutterlose, J. & Schweigert, G. Belemnite ecology and the environment of the Nusplingen Plattenkalk (Late Jurassic, southern Germany): evidence from stable isotope data. *Lethaia* **47**, 512–523 (2014).
32. Viohl, G. Die lithographischen Plattenkalke im engeren Sinne. *Solnhofen—Ein Fenster in die Jurazeit. München, Verlag Dr. Friedrich Pfeil* 78–100 (2015).
33. Hoffmann, R., Stevens, K., Keupp, H., Simonsen, S. & Schweigert, G. Regurgitalites – a window into the trophic ecology of fossil cephalopods. *JGS* **177**, 82–102 (2020).
34. Serafini, G., Gordon, C. M., Foffa, D., Cobianchi, M. & Giusberti, L. Tough to digest: first record of Teleosauroidea (Thalattosuchia) in a regurgitalite from the Upper Jurassic of north-eastern Italy. *Papers in Palaeontology* **8**, e1474 (2022).
35. Broili, F. Beobachtungen an Pterodactylus. *Sitzungsberichte der mathematisch-physikalischen Klasse der Bayerischen Akademie der Wissenschaften München* 139–154 (1938).
36. Janicke, V. & Schairer, G. Fossilerhaltung und Problematica aus den Solnhofener Plattenkalken. N Jb Geol Paläont. *Mh* **1970**, 452–464 (1970).
37. Janicke, V. Ein *Strobilodus* als Speiballen im Solnhofer Plattenkalk (Tiefes Untertithon, Bayern). *Neues Jahrbuch für Geologie und Palaeontologie* **1970**, 61–64 (1970).
38. Wellnhofer, P. Die Pterodactyloidea (Pterosauria) der Oberjura-Plattenkalke Suddentschlands, Bayer. *Akad. Wiss. Math. Nat. Klasse Nf. H* **141**, 1–133 (1970).
39. Barthel, K. W. *Solnhofen: Ein Blick in Die Erdgeschichte*. (Ott, 1978).

40. Bennett, S. C. A new specimen of the pterosaur *Scaphognathus crassirostris*, with comments on constraint of cervical vertebrae number in pterosaurs. *Neues Jahrbuch fur Geologie und Palaontologie-Abhandlungen* **271**, 327–348 (2014).
41. Witton, M. P. Pterosaurs in Mesozoic food webs: a review of fossil evidence. in *New Perspectives on Pterosaur Palaeobiology* (eds Hone, D. W. E., Witton, M. P. & Martill, D. M.) vol. 455 0 (Geological Society of London, 2018).
42. Sanz, J. L. *et al.* An Early Cretaceous pellet. *Nature* **409**, 998–1000 (2001).
43. Zheng, X. *et al.* Exceptional dinosaur fossils reveal early origin of avian-style digestion. *Scientific Reports* **8**, 14217 (2018).
44. O'Connor, J. K. *et al.* A new enantiornithine (Aves: Ornithothoraces) from the Lower Cretaceous Xiagou Formation with unusually short pubes. *Geobios* **90**, 123–131 (2025).
45. Neumann, C. Evidence of predation on Cretaceous sea stars from north-west Germany. *Lethaia* **33**, 65–70 (2000).
46. Bishop, G. A. Traces of Predation. in *The Study of Trace Fossils: A Synthesis of Principles, Problems, and Procedures in Ichnology* (ed. Frey, R. W.) 261–281 (Springer Berlin Heidelberg, Berlin, Heidelberg, 1975). doi:10.1007/978-3-642-65923-2_13.
47. Hattin, D. E. Fossilized regurgitate from Smoky Hill Member of Niobrara Chalk (Upper Cretaceous) of Kansas, USA. *Cretaceous Research* **17**, 443–450 (1996).
48. Everhart, M. Evidence of feeding on mosasaurs by the Late Cretaceous lamniform shark, Cretoxyrhina mantelli. *Journal of Vertebrate Paleontology* **17**, 43A-44A (1999).
49. Everhart, M. J. First records of plesiosaur remains in the lower Smoky Hill Chalk Member (Upper Coniacian) of the Niobrara Formation in western Kansas. *Transactions of the Kansas Academy of Science* **106**, 139–148 (2003).

50. Everhart, M. Plesiosaurs as the food of mosasaurs; new data on the stomach contents of a *Tylosaurus proriger* (Squamata; Mosasauridae) from the Niobrara Formation of western Kansas. *The Mosasaur* **7**, 41–46 (2004).
51. Capasso, L. Palaeontological evidence of piscivorous habits of some pycnodonts from the middle Cenomanian of Lebanon. **41**, 91–110 (2019).
52. Freimuth, W. J., Varricchio, D. J., Brannick, A. L., Weaver, L. N. & Wilson Mantilla, G. P. Mammal-bearing gastric pellets potentially attributable to *Troodon formosus* at the Cretaceous Egg Mountain locality, Two Medicine Formation, Montana, USA. *Palaeontology* **64**, 699–725 (2021).
53. Muzzopappa, P., Martinelli, A. G., Garderes, J. P. & Rougier, G. W. Exceptional avian pellet from the Paleocene of Patagonia and description of its content: a new species of calyptocephalellid (Neobatrachia) anuran. *Papers in Palaeontology* **7**, 1133–1146 (2021).
54. Wilson, M. V. H. Paleoecology of Eocene lacustrine varves at Horsefly, British Columbia. *Can. J. Earth Sci.* **14**, 953–962 (1977).
55. Wilson, M. V. H. Middle Eocene freshwater fishes from British Columbia. *Royal Ontario Museum, Life Sciences Contributions* **113**, 1–61 (1977).
56. Wilson, M. V. H. Eocene lake environments: Depth and distance-from-shore variation in fish, insect, and plant assemblages. *Palaeogeography, Palaeoclimatology, Palaeoecology* **32**, 21–44 (1980).
57. Wilson, M. V. H. Predation as a source of fish fossils in Eocene lake sediments. *Palaios* **2**, 497–504 (1987).
58. Buskirk, B., Hunt, A. & Lucas, S. Who's eating who? Preliminary analysis of enigmatic bromalites from the Eocene Florissant Formation, Colorado. in vol. 47 346 (2015).
59. Mayr, G. & Schaal, S. Gastric pellets with bird remains from the early Eocene of Messel. *PALAIOS* **31**, 447–451 (2016).

60. Gunnell, G. *et al.* Ferae—Animals that eat animals. *Messel—An Ancient Greenhouse Ecosystem; Smith, KT, Schaal, SFK, Habersetzer, J., Eds* 270–283 (2018).

61. Smith, K., Čerňanský, A., Scanferla, A. & Schaal, S. Lizards and snakes-Warmth-loving sunbathers. in 123–147 (2018).

62. Murphey, P. C., Torick, L. L., Bray, E. S., Chandler, R. & Evanoff, E. Taphonomy, Fauna, and Depositional Environment of the Omomys Quarry, an Unusual Accumulation From the Bridger Formation (Middle Eocene) of Southwestern Wyoming (USA). in *Eocene Biodiversity: Unusual Occurrences and Rarely Sampled Habitats* (ed. Gunnell, G. F.) 361–402 (Springer US, Boston, MA, 2001). doi:10.1007/978-1-4615-1271-4_15.

63. Hunt, A. & Lucas, S. Cenozoic vertebrate trace fossils of North America: Ichnofaunas, ichnofacies and biochronology. *New Mexico Museum of Natural History and Science Bulletin* **42**, 17–41 (2007).

64. Lucas, S., Emry, R., Krainer, K., Hunt, A. & Spielmann, J. Strigilites (fossilized own pellets) from the Oligocene of Wyoming. *New Mexico Museum of Natural History and Science* **Bulletin 57**, 325–336 (2012).

65. Engesser, B. & Storch, G. Eomyids (Mammalia, Rodentia) from the Lake Oligocene of Enspel, Westerwald (West Germany). *Eclogae Geologicae Helvetiae* **92**, 483–493 (1999).

66. Smith, K. T. & Wuttke, M. Avian pellets from the late Oligocene of Enspel, Germany—ecological interactions in deep time. *Palaeobiodiversity and Palaeoenvironments* **95**, 103–113 (2015).

67. Gawne, C. E. Rodents from the Zia Sand Miocene of New Mexico. American Museum novitates; no. 2586. (1975).

68. Álvarez-Parra, S. *et al.* The early Miocene lake of Foieta la Sarra-A in eastern Iberian Peninsula and its relevance for the reconstruction of the Ribesalbes–Alcora Basin palaeoecology. *Acta Palaeontologica Polonica* **66**, s013–s030 (2021).

69. Nasif, N., Esteban, G. & Ortiz, P. Novedoso hallazgo de egagrópilas en el Mioceno tardío, Formación Andalhuala, provincia de Catamarca, Argentina. *Serie correlación geológica* **25**, 105–114 (2009).

70. Walton, A. H. Owl pellets and the fossil record. in *Evolutionary Paleobiology of Behavior and Coevolution* (ed. Boucot, A. J.) 233–241 (Elsevier, Amsterdam, 1990).

71. Czaplewski, N. J. An owl-pellet accumulation of small Pliocene vertebrates from the Verde Formation, Arizona, USA. *Palaeontologia Electronica* **14**, 33 (2011).
